# A systematic review of providers’ experiences of facilitating group antenatal care

**DOI:** 10.1186/s12978-021-01200-0

**Published:** 2021-09-07

**Authors:** Jalana Lazar, Laura Boned-Rico, Ellinor K. Olander, Christine McCourt

**Affiliations:** City, University of London, London, UK

**Keywords:** Group antenatal care, Prenatal care, Maternity care professionals, Providers’ experiences

## Abstract

**Background:**

Group antenatal care is a rapidly expanding alternative antenatal care delivery model. Research has shown it to be a safe and effective care model for women, but less is known about the perspectives of the providers leading this care. This systematic review examined published literature that considered health care professionals’ experiences of facilitating group antenatal care.

**Methods:**

Systematic searches were conducted in seven databases (Cinahl, Medline, Psychinfo, Embase, Ovid Emcare, Global Health and MIDRS) in April 2020. Qualitative or mixed methods studies with a significant qualitative component were eligible for inclusion if they included a focus on the experiences of health care providers who had facilitated group antenatal care. Prisma screening guidelines were followed and study quality was critically appraised by three independent reviewers. The findings were synthesised thematically.

**Results:**

Nineteen papers from nine countries were included. Three main themes emerged within provider experiences of group antenatal care. The first theme, ‘Giving women the care providers feel they want and need’, addresses richer use of time, more personal care, more support, and continuity of care. The second theme, ‘Building skills and relationships’, highlights autonomy, role development and hierarchy dissolution. The final theme, ‘Value proposition of group antenatal care’, discusses provider investment and workload.

**Conclusions:**

Health care providers’ experience of delivering group antenatal care was positive overall. Opportunities to deliver high-quality care that benefits women and allows providers to develop their professional role were appreciated. Questions about the providers’ perspectives on workload, task shifting, and the structural changes needed to support the sustainability of group antenatal care warrant further exploration.

**Supplementary Information:**

The online version contains supplementary material available at 10.1186/s12978-021-01200-0.

## Plain language summary

Receiving antenatal care in a group setting has been found to be safe and satisfying for women and is supported by international public health guidelines. However, questions remain about the experience of health care professionals tasked with providing this model, such as whether they like working in this model and whether they support its expansion. To answer these questions, the team searched for studies about the experiences of health care providers with group antenatal care, and only included those studies where providers themselves spoke about their own experiences of providing this kind of care. Our review demonstrated that midwives, doctors, nurses and community health workers mostly enjoyed facilitating group antenatal care. They particularly appreciated the ability to give women the kind of care they felt women want and need. Health care providers also experienced some changes in their professional roles, in relation to both the women they serve and their colleagues and organizations. In order to determine if group antenatal care models are a satisfying and sustainable option for health care professionals in the long term, more research is needed.

**Supplementary Information:**

The online version contains supplementary material available at 10.1186/s12978-021-01200-0.

## Resume

**Contexte:**

Les sessions prénatales en groupe constituent une approche alternative en pleine expansion. La recherche a montré qu'il s'agissait d'une approche aux sessions prénatales qui est sûre et efficace pour les femmes. Cependant, on en sait moins sur les perspectives des prestataires qui dirigent ces sessions. Cette revue systématique a examiné la littérature publiée qui tenait compte des expériences des professionnels de la santé en matière de facilitation des sessions prénatals en groupe.

**Méthodes:**

Des recherches systématiques ont été menées dans sept bases de données (Cinahl, Medline, Psychinfo, Embase, Ovid Emcare, Global Health et MIDRS) en avril 2020. Les études qualitatives ou mixtes avec une composante qualitative significative étaient éligibles pour l'inclusion si elles portaient sur les expériences des prestataires qui avaient facilité les sessions prénatales en groupe. Les directives de dépistage Prisma ont été suivies et la qualité des études a été évaluée de manière critique par trois examinateurs indépendants. Les résultats ont été synthétisés par thème.

**Résultats:**

Dix-neuf articles de neuf pays ont été inclus. Trois thèmes principaux sont ressortis des expériences des prestataires de sessions prénatales en groupe. Le premier thème, « Donner aux femmes les soins qu'elles désirent et dont elles ont besoin », porte sur une utilisation plus riche du temps, de soins plus personnels, d'un plus grand soutien et de la continuité des soins. Le deuxième thème, « Construire des compétences et des relations », met l'accent sur l'autonomie, le développement des rôles et la dissolution de la hiérarchie. Le dernier thème, « La valeur ajouté des sessions prénatales en groupe », traite sur l'investissement des prestataires et de leur charge de travail.

**Conclusions:**

L'expérience des prestataires de soins de santé en matière de prestation de sessions prénatales en groupe était globalement positive. Les opportunités d’offrir des soins de haute qualité qui profitent aux femmes tout en permettant aux prestataires de développer leur rôle professionnel ont été appréciées. Les questions sur les points de vue des prestataires sur la charge de travail, le transfert des tâches et les changements structurels nécessaires pour soutenir la durabilité des sessions prénatales de groupe méritent une exploration plus approfondie.

## Background

Group antenatal care (GANC) models have been recognized by the World Health Organization (WHO) as a health system innovation that may help achieve the global goals of a positive pregnancy experience for every woman and an end to preventable maternal deaths by improving access, attendance and continuity and quality of care [1].

Typically, GANC models provide clinical risk assessment, education and support (the essential elements of antenatal care) in a group setting of pregnant women with similar gestational ages, and the care is facilitated by the same healthcare provider throughout the pregnancy course. Where resources allow there are two group leaders, one of whom must be a clinical antenatal care provider, and this is most often a midwife. The most widely researched model of GANC, Centering® Pregnancy, was developed by a midwife and outlines 13 essential elements to successful GANC, and has been implemented in the U.S., Canada, Australia and the Netherlands [2]. It has also been adapted to meet the context and needs of low- and middle-income countries (LMICs) [3]. Other bespoke models have been developed in both high- and low-income countries. Globally, all models tend to include a relatively stable group of pregnant women meeting in a group space, performing self-assessment checks and having extended face-to-face time with a provider in a facilitative fashion that prioritizes peer-to-peer learning and support [3–5]. GANC visits follow the national standard antenatal care schedules, yet allow women 15–20 face-to-face hours with the same antenatal care provider as opposed to the current traditional care average of two and half hours of time with a provider (who may not always be the same) [6–11].

Since the first pilot GANC programmes began in 1994, research has shown that women like this model of care. High satisfaction is demonstrated across multiple studies in high-, middle- and low-income countries (particularly among vulnerable populations), and attendance rates are higher than with traditional antenatal care [7–9, 11–13]. In addition to being a satisfying model of care, the outcomes for mothers and babies in GANC are at a minimum comparable in outcomes to traditional care models, and some studies have shown that GANC improved birth outcomes, in particular among African Americans and Latinas in the United States, as well as in trials in Iran, Nigeria and Kenya [8, 14–19].

The theoretical grounding for the GANC model is, as yet, unclear, and remains underdeveloped [20]. The effects of GANC are most likely multi-factorial and involve both individual theories of caring, trust, empowerment and self-efficacy and broader group mechanisms related to peer and societal support [21–25]. Logically, the health care providers facilitating group care have an impact on its efficacy, but the mechanisms of effect are, as yet, undefined.

GANC is unique in that it is a group, not a class. Rather than a didactic hierarchical information transfer, the model was conceived as a sharing of experience and knowledge guided by professionals in a facilitative fashion. Research has shown that outcomes are better with model fidelity [26], which implies the possibility that skilled facilitation improves the antenatal care environment. The Centering® Pregnancy model was originally conceived as being ‘ideally, group care led by a CNM/CM [Certified Nurse Midwife/Certified Midwife] or nurse practitioner skilled in group process. An additional person, a nurse or aide, will facilitate the flow of the group and help with any follow-up necessary' [27 p 48]. Rising, Kennedy and Klima [28] also posited the midwifery model of care as a theoretical framework for understanding the success of group care. Shared decision-making, listening to women and a focus on the contribution of women and building partnerships are all characteristics of midwifery care. These skills may predispose midwives to find facilitative care more intuitive than physicians do [29], but group facilitation is a learned skill that even midwives may find challenging [30].

Although the original conception of GANC had midwives leading, there has also been interest and research on physician-led groups [3, 31–33]. There is no published literature on groups led by other health care or social work professionals at this time. The model also provides a unique opportunity for interprofessional collaboration, particularly in the case of women with complicated conditions or in under-resourced areas where community health workers play an important outreach role [34, 35].

As provider buy-in is essential to successful implementation of GANC [29], and as it is recommended that two clinical professionals lead group care models, and given the endorsement of midwives as recommended antenatal care providers globally [36], the questions arise; who is currently providing GANC? and what has been their experience of providing this innovative model of care? A Cochrane review by Catling et al. [37] attempted to look at provider satisfaction and found no data with which to examine their question. Several articles have examined provider views on GANC as part of pilot or feasibility studies. Where providers are presented with information and demonstrations of the model, they seem enthusiastic about the possible benefits of the model but also highlight potential personal and professional obstacles [33, 38, 39].

GANC has been the subject of research for over two decades now, and given the global pivot towards midwifery models of care and continuity of care, as well as the context of global maternity care staffing shortages and evidence of dissatisfaction and burnout among care providers with current ways of working [40], a systematic review foregrounding providers’ insights on facilitating GANC is timely. The aim of this review is to explore the experiences of the providers who have themselves facilitated GANC, as their input is a critical component in further successful expansion and integration of GANC.

## Methods

The protocol for this review was registered in PROSPERO, reference CRD42020171848.

### Searching

After consultation with a health sciences librarian, searches were performed by JL in seven databases: Cinahl, Medline, Psychinfo, Embase, Ovid Emcare, Global Health and MIDRS. Hand searching and the Scopus database was used to identify further citations from relevant publications, in addition to a complete review of the bibliographies of the Centering® Healthcare Institute and Group Care Global [41, 42]. OpenGrey was also reviewed for any pertinent grey literature. The search was date limited from January 1990 through April 2020 to correspond with the development and implementation of group care models. Search terms chosen related to group antenatal care, health care professionals and experiences. Search terms are listed in Additional file 1.

### Screening

Inclusion and exclusion criteria are listed in Table 1. Papers were included if they contained qualitative data relating to the experiences of health care providers facilitating GANC or group antenatal plus postnatal care; this included mixed methods studies as well as qualitative studies. GANC was defined for the purposes of inclusion as any antenatal care with a clinical component that comprises more than four women meeting in a group. As the focus of this review is on the experience of facilitating GANC, reviewers excluded papers in which it was unclear whether the participants had facilitated groups themselves (the reviewer contacted study authors where possible to make this determination); studies in which providers speculated on facilitation of GANC; and studies that did not report experiences from the viewpoint of the health care provider.

**Table 1:** Study inclusion and exclusion criteria

	Inclusion	Exclusion
Participants	All healthcare providers who have facilitated group antenatal care where group antenatal care is defined as: defined as any antenatal care with a clinical component that includes more than four women meeting in a group	Studies of GANC with no health care provider views and perspectives Studies where it cannot if the participants themselves facilitated the GANC will be excluded
Phenomenon of interest	The focus will be on the experiences and perspectives of health care providers (physicians, midwives, nurses, allied health professionals) who have been involved with facilitation of group antenatal care (GANC) models	Any studies which describe the experience of women with their health care provider in group antenatal settings will not be included unless it is described from the HCP point of view
Outcomes	This review will seek to understand the experiences of health care providers as it pertains to the acceptability, feasibility, and sustainability of group models of care in diverse healthcare systems	Outcomes related to women
Study design	Study must have a qualitative component Mixed method studies that include a relevant qualitative component in the findings	Studies collecting data quantitatively only
Study focus	Studies should focus on experience of facilitating/participating in group antenatal care	Focus on women
Setting	All countries	None

**Fig. 1:**
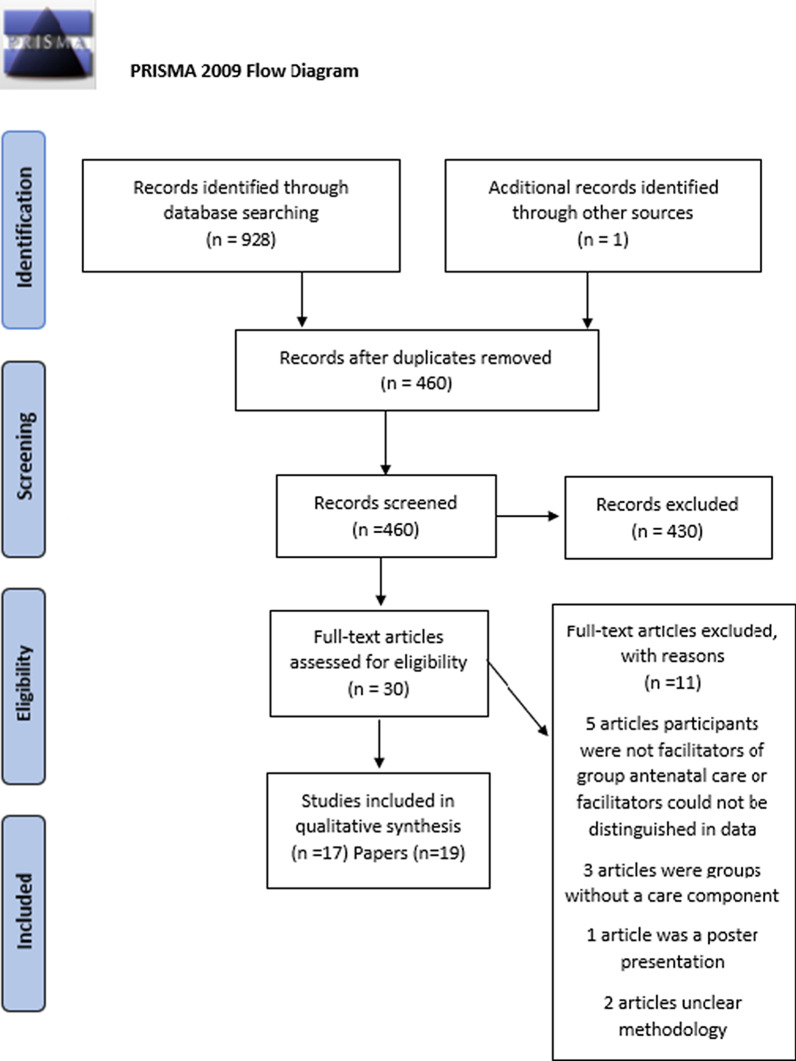
Search statistics prisma flow diagram [43]

The search and screening process followed the Prisma guidelines [43] (see Fig. 1.) All retrieved studies were imported into Refworks for deduplication and then into Rayyan software for screening [44]. One reviewer (JL) screened by title and abstract for relevance to the review topic, and 20% of those were double screened by a second reviewer (LBR) to ensure reliability. The full texts of all relevant studies were screened by both JL and LBR against the inclusion criteria, and conflicts regarding inclusion were resolved in consensus with two other members of the review team (CMC and EO).

### Quality appraisal

The methodological rigour of all included studies was appraised using the Critical Appraisal Skills Programme tool for qualitative research [45]. Reviewers (JL, LBR, CMC) independently rated the papers high, medium or low quality and discussed and noted discrepancies, but study quality did not exclude papers from the review as there was rich data to be found in some studies of lower quality. CMC was not involved in any evaluations of her own publications.

### Data extraction

See Table 2 for data extracted from each study. This included study author and date, type of participant health care professional (e.g. physician, midwife), study location, study design and methodology of qualitative data collection, and key findings (with particular reference to experiences of providers).

**Table 2:** Table of data extracted from included studies

Study first author, year	Country	Study aims	Participant, setting	Study design, data collection, and analysis	Quality	Findings	Collaborators
Allen, J., 2015 [51]	Australia	Examine younger women’s experiences of caseload midwifery incorporating gANC	4 Midwives Caseloading practice for women under 21 Purposive sampling	Qualitative critical ethnography FGD and observations thematic analysis starting with women's data and applied to midwives	H	Women had some benefits, and midwives observed some benefits for participants. The conclusion was that the model interfered with relationship building	Midwives co-facilitate with each other
Baldwin, K., 2011 [52]	USA	Midwives’ thoughts, feelings, perceptions from pre-implementation through facilitation of five sessions of CP, also focus on sustainability	6 Midwives 5 clinics in different regions of the United States (Northeast, Midwest, South) recruited at CP training Convenience sampling	Qualitative Design SSI face to face and over telephone at 5 different time periods transtheoretical health education model Colazzis method and thematic analysis	H	Emergence of five themes progression fromcurrent practice is just fine through anxiety about the model to empowerment and looking to the future	Midwives co-facilitate with each other
Barnes, J., 2016 [53]	UK	Evaluation of the feasibility of the group family nurse partnership (FNP) program	8 Family nurse partnership nurse midwives 4 community midwives 4 supervisors 4 family support workers Purposive sampling	Mixed Methods FGD and SSI Content Analysis	M	Content and format was positive for participants and FNP facilitators but women struggled to attend regularly and most vulnerable were not recruited and FNP found working with community staff challenging	FNP midwives cofacilitated with community midwives or family support workers
Craswell, 2016 [54]	Australia	Evaluate a group care model collaboration between an academics, students, and public health service midwives	5 Midwives 5 midwifery students clinic held on university grounds Purposive sampling	Qualitative design SSI and FGD thematic analysis following donobedians structure process outcome framework	H	Positive opportunity for continuity of care for midwifery students and positive collaboration between university and clinic midwives and positive views from participants	Academic midwives co-facilitate with students and clinic midwives
Hunter, L., 2018 [67]	UK	Feasibility of implementing gANC in high diversity area by exploring midwife and other maternity care provider views	16 Stakeholders 9 facilitating midwives 1 student midwife large diverse London NHS trust Purposive sampling	Inductive qualitative approach SSI informal group discussions and workshop post implementation the matic analysis	H	Intervention was supported as a solution to dissatisfaction with standard care, worries about privacy, self-checking and partners were overcome with adequate support and training and experience with the model and midwives enjoyed delivering care this way and felt satisfied with that care	Midwives co-facilitate with each other
Klima, C., 2009 [63]	USA	Feasibility of implementing CP in a large urban clinic and associated outcomes	4 Midwives 5 health centre staff Large urban public health clinic Purposive sampling	Mixed methods feasibility FGD thematic analysis	M	Midwives and staff felt women enjoyed their care and improved their attendance and satisfaction midwives and staff experienced challenges with implementation aspects such as scheduling and midwives found facilitation challenging and losing one to one interaction	Midwives co facilitate with project assistant or medical staff (training undefined)
Lori, J., 2016 [55]	Ghana	Does gANC improve providers perceptions of communication and engagement-does facilitative gANC improve health information delivery-is a health literacy skills framework suitable for maternal health literacy development	6 Midwives (4 participated in FGD) 1 nurse who co-facilitated groups busy clinic Ashanti region Convenience sampling	Mixed methods survey and FGD constant comparative analysis	H	No significant difference in survey of communication and engagement focus group identified themes of improved understanding of patient concerns, enhanced information and sharing with facilitated discussion, and improved communication with picture cards	Midwives co-facilitated with each other and a support nurse
Lundeen, T., 2019 [60]	Rwanda	Understand the experience and job satisfaction and perceived stress of gANC providers as compared to standard ANC providers	59 Nurses and midwives completed questionnaire 29 participated in FGD 18 health centres in Rwanda Cluster randomized sampling	Mixed methods nested study survey 3 FGD thematic analysis	H	Survey showed no change in job satisfaction or perceived stress however 86% midwives said they preferred gANC and FGD showed benefits for women and midwives and opportunities for problem solving implementation challenges with peer nurses and midwives	Midwives and nurses co-facilitate with CHWs whose experiences were not reported in this article
Maier, B., 2013 [62]	Australia	Reflection piece	1 Midwife caseloading Large urban hospital	Personal reflection	L	Author found this a very satisfying way to deliver antenatal care and thus extended it to postnatal groups and included students	Doesn't mention a co-facilitator but did have midwifery students in group
McDonald, S., 2014 [56]	Canada	Experiences of low-risk women and their care providers with gANC	5 Midwives Midwifery clinic in Ontario Purposive sampling	Qualitative descriptive study FGD thematic analysis	H	Women felt they received more information and support but less one on one time with midwifemidwives saw systems level challenges but saw professional benefits such as reduced workload and more autonomy for women	Midwives co-facilitate with each other
McNeil, 2013 [58] Vekved, 2017 [59]	Canada	Understand the central meaning of centering pregnancy to family physician facilitators and perinatal educator facilitators	3 Family physicians providing CP care in Calgary 5 perinatal educators providing CP care Low-risk group practice in Calgary Purposive sampling	Phenomenological approach IDI meaning units/thematic analysis confirmation fgd and interviews and re-analysis	M/H	Core meaning for physicians of "providing richer care" examined across six themes around more time and more satisfaction and seeing women create relationships with each other and physicianperinatal educators found a core meaning of "invested in success" covered by six themes including bridging the gap and getting to knowing and stepping back	Physicians co-facilitate with perinatal educators
Novick, 2013 [49] Novick, 2012[66]	USA	What are perceived as the challenges to implementing centering and how is centering model adapted to meet these challenges?	2 Nurse midwife group leaders 3 support staff included in participant observations 2 urban clinics in north-eastern US Purposive sampling	Longitudinal qualitative study interpretive description (Thorne, 2008) SSI with group leaders participant observation of centering sessions thematic analysis and situational mapping	M/M	Leaders were committed to gANC but hampered by resource constraints which resulted in modifications to the model that further impacted successgroup leaders felt strongly benefits to vulnerable women of participating in this model of care and women participating in this group found some respite from their stressors	One midwife had a staff member co-facilitator (not identified) the other had no co-facilitator
Novick, G., 2015 [50]	USA	Identify barriers and facilitators to implementing CP in 6 urban sites	14 Clinical site staff ( 2 administrators, 4 obstetricians, 3 nurse midwives, 1 registered nurse, 3 social workers, and 1 dietician) of whom 6 facilitated care Urban women’s health care clinics in 6 large hospitals Purposive sampling	Qualitative research conducted alongside a cluster RCT IDI and SSI A priori coding and implementation frameworks ATLAS software	H	Thriving sites had organizational cultures that supported innovation and committed staff and provider champions	Some had co-facilitators but they are not specified
Patil, C., 2013 [64]	Malawi/ Tanzania	Determine if CP is an acceptable model in African antenatal care contextdevelop CP curriculum that maintains national guidelines and essential CP elementssmall pilot trial in Malawi	1 Administrator 6 midwives 4 HSAs (community health workers)	Feasibility study with small pilot in advance of RCT ethnographic rapid assessment (action research model) observations and field notes by researchers of groups FGD with semi structured guide	H	Centering Pregnancy Africa was feasible and acceptable in the Malawian context and midwives adapted to and enjoyed the facilitation and greater information sharing	Co-facilitation format not specified
Teate, 2013 [57]	Australia	Explore midwives’ experiences as they moved from one-to-one care to Centering Pregnancy care	8 Midwives 2 public maternity services in Sydney (3 antenatal clinics, 2 community health centres) Purposive sampling	Qualitative descriptive and iterative action research designpre- and post-surveys, checklists, FGD, observations of facilitation meetings thematic content analysis	H	Midwives progressed throughout the action research from initial anxiety through to appreciating the benefits of CP for women and for their own relationship with women and for the support and training they received	Midwives co-facilitated with each other
Thapa, P., 2019 [61]	Nepal	# of ANC visitinstitutional birth rate experience of the model and mechanism of impact from a variety of perspectives	2 CHW and 2 government care providers Rural Nepal Purposive sampling (one interview with gov't care provider excluded)	Mixed methods cluster-controlled trial FGD with participants KII with providers directed content analysis approach theory of change codes and moving on to open coding [p. 4 Qualitative data were only gathered from those with direct experience of the intervention supervisory and Nyaya program staff had insights-where to include]	M/H	Women appreciated groups for learning and support providers appreciated relationship with community health workers and birth planning was a challenge for women and facilitators	Government midwife co-facilitated with Nyaya health chw
Wisanskoonwong, P., 2011 [65]	Thailand	Develop a culturally appropriate model of group antenatal care for Thai women	1 Midwife Meeting room near antenatal clinic of large hospital in Bangkok	Feminist Action researchpersonal reflection and evaluation	M	Reflection on decision to not wear her uniform for group care resulted in her perception of more equalized relationships in group care and giving up role of expert allowing more open discussion	Doesn’t mention co-facilitator in reflection

*FGD* focus group discussion, *SSI* semi-structured interview, *KII* key informant interview, *CP* centering pregnancy

### Data analysis and synthesis

The full text of the results section, including participant quotations verbatim, was uploaded into NVivo 11 software. Then following Thomas and Harden’s [46] approach to thematic analysis, the results section of each study was coded line by line and descriptively by one reviewer (JL), and then organized into subthemes that had reciprocal meaning across studies, whilst attempting to preserve faithfulness to the experiences of participants in the individual studies [47] and taking care to include meanings that refuted one another [48]. The organization of the subthemes into overarching themes then pushed the analysis beyond translation into interpretation in order to add new concepts and meaning whilst remaining aligned with the original findings [46]. The themes and subthemes were discussed amongst three reviewers (JL, CMC, EO) to ensure accurate reflection of individual study findings and maintain relevance to the aims of this review.

## Results

### Included studies

A total of 928 studies were identified through electronic database searching with an additional study identified through hand searching of citations. After duplicates were removed and screening was completed (see Fig. 1), 19 papers from 17 studies were included. Five papers were from LMICs and the remaining studies were from high-income countries. Two papers were personal reflections of midwives conducting group care; 10 papers were pure qualitative research; and the remaining papers were mixed methods analysis that included a qualitative component. In Rwanda, Nepal and one of the U.S. studies, the qualitative analysis was conducted alongside a cluster randomized control trial. Eleven papers were assessed as being high quality, one as medium–high quality, six as medium quality and one as low quality. The low-quality paper was a personal reflection of an Australian midwife’s direct experience of facilitating GANC and thus, although lacking methodological rigour, was clearly relevant to the research question. The vast majority of the facilitating providers were midwives (n = 133); in the Rwandan study, both midwives and nurses (n = 59) were facilitating and no distinction was made between them in the focus group discussions. The other providers facilitating included three family practice physicians, five perinatal educators and four family support workers. Two papers mentioned ancillary medical staff (qualifications not specified) and obstetricians. In some cases, it is unclear from the papers what facilitative role, if any, the medical staff and obstetricians had [49, 50]. In seven studies midwives co-facilitated with other midwives [51–57], sometimes from academic backgrounds or different disciplines, and in one case with the aid of a support nurse. In one study physicians facilitated with perinatal educators [58, 59]. In two studies midwives or nurses worked with community health workers [60, 61]. In two papers midwifery students were involved in the facilitation process [54, 62]. The remaining studies had either no co-facilitator or did not describe a co-facilitator. Some mentioned ancillary medical staff or programme staff but didn’t specify their training or participation in the facilitation process [49, 50, 63–65] (see Table 2.)

### Qualitative themes

**Fig. 2:**
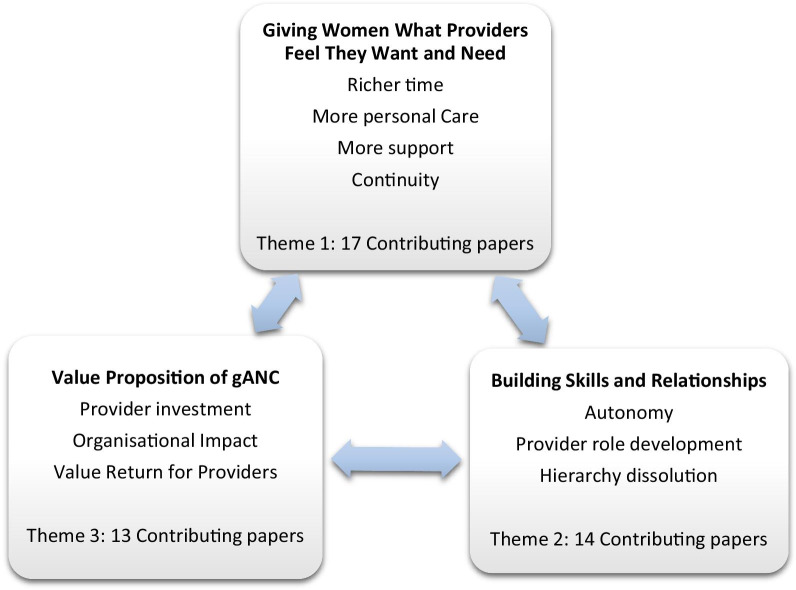
Themes of the review

Three overarching themes emerged from the analysis of provider experiences with facilitation of GANC. Firstly, the experience of providing the elements of care they know women want; secondly, the experience of skill building and role change; and thirdly, the theme entitled Value Proposition of GANC’ addressing provider investment and workload (see Fig. 2).

#### Giving women what providers feel they want and need: the satisfying experience of giving women personalized, supportive, high-quality care

In a GANC model, providers experience the opportunity to offer women many of the attributes of care that influence their uptake and satisfaction with antenatal care.

Now due to this program pregnant women are also enjoying it a lot. Now pregnant women come and ask us, ‘When are we coming for our next checkup? When are we going next?’ They ask this and then when they get to sit in a group … Now they don’t have the ‘aa, why do we need to go for checkup?’ kind of mentality.—Community Health Worker in Nepal [61, p 10]

Providers uniformly related that women who participated in group care were happier and seemed to want to come for prenatal care. They stated that women also appreciated not having to wait for their visits, a common issue in this crowded clinic.—Clinicians in the US [63, p 30]

The following subthemes describe providers’ experiences of providing care that women want through the richer use of time, more depth in the time allotted, more personalized care, more supportive care and continuity of care.

##### Richer use of time

An adequate quantity and quality of time in antenatal care has repeatedly been identified as a key component of what women want, and what providers themselves often feel they lack. In this review, providers repeatedly commented on the ways in which the time was spent in GANC was more productive [66]. The richer use of time was facilitated by decreased repetition and the ability to achieve more educational and personal depth of care in the time allotted in group care as compared to standard antenatal care [55, 57, 58, 64]. The restructuring of provider hours with group models afforded providers more time to deliver higher quality care.

In our regular clinic…sometimes we’re kind of rushed and moving pretty quickly and so [I like] to just feel like we can sit down and get in depth with people. … I like that. … I’d rather have a thick novel than a one paragraph of a magazine article.—Physician in Canada [58, p 4]

##### More personalized care

Providers appreciated that the extra time spent in discussion in GANC models allowed them to assess women’s knowledge and better meet their needs, in some ways offering care that is more personalized than in standard care.

…facilitating midwives felt that GANC enabled them to be truly ‘with woman’, building up trust and rapport over multiple encounters and addressing social, emotional, and clinical needs: It’s not one-to-one but honestly, I can remember all of the women’s names and you can’t really say that for when you are in an antenatal clinic and all the women come in and out, you don’t remember them.—Midwife in the U.K. [67, p 61]

In addition to getting to know women better, GANC allowed providers more possibility to tailor their care and listen and respond to feedback from numerous women and other providers. The additional opportunities to ask and answer questions invested the time spent with richer education and support around pregnancy and parenting [58, 60, 63].

In the past, pregnant women used to come and listen to a brief talk from the nurse. But today, they come and sit together with the nurse and share. They ask questions and get answers to them. In the past, the nurse could fail to get time to answer to their questions; so they could go back home without answers. Today, they are free to ask whatever they want; they feel at ease with the nurse; they behave like friends.—Nurse/Midwife in Rwanda [60, p 8]

Midwives also commented that the increased feedback and communication made their jobs faster and easier [55].

##### More supportive care

Providers facilitating GANC appreciated the peer component as a vital element that engendered a supportive environment, normalized the pregnancy experience and enabled health behaviour changes.

They witnessed the creation of a community, and saw transformative support for young or vulnerable members and bonding between women with the exchange of personal details and valuable information that filled important knowledge and support gaps [55, 56, 58].

…sometimes there’s sort of synchrony in the life issues that the women are having in terms of relationships, particularly with their partners. They teach each other and they teach me about ways in which they are able to cope, and demonstrate some strength in their lives, no matter how chaotic sometimes it appears or how crazy it is.—Midwife in the U.S. [66, p 598]

Additionally, normalization of pregnancy as a healthy state in the presence of peers was identified as an important reassurance for women and a validation of provider beliefs [56, 67]. Maternity care providers identify the group setting as being an advantageous way for women to transition knowledge into healthy behaviours, where sharing experiences among peer experiences in the presence of a clinical facilitator was a motivator for health-seeking behaviour and health-promoting behaviours [58–61, 64, 65]. Providing care that supported knowledge acquisition and behaviour change was satisfying for providers,

As for me, this group care program has pleased us very much; you can even learn of this fact through much excitement of the group members. For us who lead group care, we can see it. You can see that mothers are thirsty for knowing all those new things. When you discuss with them and when you are making conclusions together with them, you find the members happy, and most of them wish never to miss out.—Nurse/Midwife in Rwanda [60, p 6]

##### Continuity of care

Continuity of care has been identified as a driver of improved outcomes for women and as an important element in women’s satisfaction with their care. For providers facilitating GANC, the continuity of care delivery was an important benefit for women [51], but also for students and the providers themselves.

It contributes because they [students] won’t see it in a hospital setting, they won’t see a same group coming at the same time, on set dates…[the women] growing as a group and shifting in their pregnancies’ how comfortable they are and sharing, hearing more than one person. So I think it contributes in changing their perception of what a pregnancy journey is…—Midwife in Australia [54, p 419]

In another study, providers identified this continuity as contributing to patient safety and ease of follow-up as well as a sense of autonomy in managing their workload [67].

#### Building skills and relationships

The second theme to emerge from the data was that of experiences around skill-building and changes in the roles of providers and participants. Fourteen papers contributed to this theme, which is further explored in three subthemes: independence/autonomy, provider role development and hierarchy shifts.

##### Independence/autonomy

Providers repeatedly commented on the increased independence/autonomy of the women in GANC.

Notably, in the study of Rwandan nurses’ and midwives’ experiences facilitating GANC, the focus group participants described ways in which a key element of GANC, the self-checking component, improved care quality by shifting health surveillance tasks to women and allowing them to take more ownership of their care.

Some providers admitted that the structure of group care visits resulted in an increase in routine assessments, especially blood pressure: “We didn’t use to test blood pressure, and the effect resulting thereof could take the lives of many women. This test is very important. [In the past] it was very possible [we did not check blood pressure] even until she gives birth. They [group care participants] can test that blood pressure themselves because they already know how to do it. When they have tested one another and found out that there is one who has a problem, they inform the nurse, and the nurse can verify and provide due assistance to the woman having the problem before the situation becomes worse. Things have become very easy.”—Nurse/Midwife in Rwanda [60, p 10]

Physicians in Canada also commented on the ways in which women became more confident and knowledgeable through checking their own blood pressure and urine [58]. In one study from the U.S., this independence was viewed differently:

Some staff complained that group prenatal care was ‘spoiling’ women for individual care because they had ‘become used to coming in, doing whatever they have to do for themselves and getting everything done instead of just sitting and waiting.’—Clinician in the U.S. [50, p 469]

In addition to restructuring the task of health surveillance, providers identified the ways in which they found that GANC restructured health education and communication with women and between women.

Seeing women so comfortable with themselves and me as a health professional was a new experience. … Compared with women experiencing normal midwifery practice in Thailand, the women in my antenatal groups were more independent and talkative. Women in Thailand are usually submissive and they generally do not have the confidence to take responsibility for their own health.—Midwife in Thailand [65, p 633–4]

Other midwives were moved by ways that participating women found coming to the group made them better mothers, and the ways that shifted the focus from the midwife to the group, or the ways GANC rebuilt trust between providers and women in communities where these relationships were strained [56, 66].

##### Provider role development through facilitation and collaboration

The growth in independence and confidence in the women coincided with a shift in the role of the provider. The facilitative role was easier for some providers than others as it required providers to cede some control over what information was given and how. This was experienced by providers in the GANC model as a process of stepping back and experiencing a sense of release from some of the pressures maternity care providers experience in the delivery of antenatal care.

It was mind-blowing just how much I could just sit back and allow the group to run itself and there was no pressure, it was just easy to facilitate this group…—Midwife in Australia [57, p e35]

The relational shift that occurred in a facilitative environment was described, as above, as a sensation of relaxation and, for many, it contributed to increased feelings of job satisfaction and provider well-being.

Most times you are chatting, you have a laugh, you are doing the work, you are accomplishing what you would do antenatal [sic] but there is a different sort of atmosphere. I find it is very relaxed.—Midwife in the U.K. [67, p 61]

It takes a little bit of the pressure off of us as well to be kind of all things to everybody. To be their midwife and their best friend and their mother…it maybe defines our clinical role a little more clearly in some respects and takes away from some of that social role.—Midwife in Canada [56, p 7]

Stepping back and giving control to the group are core distinctions between didactic and facilitative interaction. This letting go and trusting the group process was not an automatic experience for providers, as demonstrated in studies that examined the experiences of providers over the course of implementing the intervention [52, 57, 64]. The fear of failing to deliver all the necessary information or being held solely accountable in a model that shares out responsibility was anxiety producing for some participants [53].

It was hard at first because…that lack of control makes you feel like, I don’t know if they’re getting the right amount of information and then I started to realize…who am I to decide what kind of information they really need?—Perinatal educator in Canada [59, p 129]

The following quote illustrates the experience of the challenges of facilitation for maternity care providers who have been trained to deliver prescribed antenatal care content. If that content is up for discussion, providers can feel that they lose control of the narrative.

It is impossible in a group to give what we give to people one-to-one because of the constraints of them [the participants] wanting to discuss it.—Family Nurse Partnership Midwife in the U.K. [53, p 178]

Providers repeatedly acknowledged anxiety about the facilitation component of GANC. They highlighted fears of being unprepared in the event the women in the group remained silent [57, 64].

As confidence in facilitation skills grew, providers experienced their groups with satisfaction. They learned how to create a comfortable environment, and use silence, encouragement, humour and guidance to create an optimal experience for participants where everyone felt equal and heard and the groups were able to create bonds and feel safe [52, 59, 65, 67]. The result was that facilitation skills made providers feel more effective.

I was able to see the group bond and work together as my skills grew.—Midwife in the U.S. [52, p 215]

Another aspect of facilitating GANC that brought about new experiences was collaborating with other professionals. This inter-provider collaboration echoed some of the peer support benefits of group care for women and worked well in instances where providers were able to play off one another’s strengths.

I learn from her [health service midwife] about the updates in clinical practice …she realises that we’re from that evidence based [approach] and so she asks for that input. She says, ‘Oh what’s the latest thinking on this? And how do you think I could do that better?’ It’s more of a discussion.—Midwife in Australia [54, p 420]

However, inter-provider collaboration could be challenging for some.

…but I have to wear the hat of the hospital midwife not the community midwife. … there has been those moments … I haven’t necessarily resonated with what the [other] midwife has said.—Midwife in Australia [54, p 419]

Inter-provider collaboration also allowed for a shift in professional hierarchies, which was the final subtheme to emerge under provider role changes.

##### Hierarchy dissolutions

GANC appeared to alter established hierarchies in antenatal care, those between pregnant women and health care providers and those between different ranks of health care professionals, such as physicians and perinatal educators or junior and senior midwives [59].

At the beginning I was ‘absolutely petrified’. Now I feel so much more confident as a midwife. I have learnt so much. It didn’t matter how junior I was to the rest of my colleagues who were also a part of it. You’ve created a relationship with them and we had fun you know, we laughed.—Midwife in Australia [57, p e35]

This hierarchy flattening was also experienced positively by providers in their relationship with the women in their groups. They found themselves more approachable and sensed the women as more open and more confident in the value they contributed to groups, and more likely to access services they might need [50, 55, 58, 64].

I am very much satisfied [with group ANC/PNC]. I would say that the success results from freedom. When we have come together, we sit and talk freely with those mothers whom we serve.—Nurse/Midwife in Rwanda [60, p 8]

The freedom in communication observed among midwives and women in the Rwandan study also occurred between midwives and managers.

I have learnt also to play a role in boldly speaking to the manager in favor of group care when elaborating the timetable. We shall inform them about how the group care activities are scheduled throughout the week so that they will provide room for the people trained to handle group care and do that very job without having much work in other services.—Nurse/Midwife in Rwanda [60, p 12]

This quote illustrates both need and desire among providers to advocate for institutional time, space, staffing and support for GANC. It speaks to the third theme that emerged from this review, which can be expressed in the unasked question of whether this model of care is worth the work, and for whom.

#### Value proposition of GANC

The third theme raised in the included studies encompassed the workload and commitment invested by providers implementing GANC, the effects of organizational support on that investment, and the value return experienced by the facilitating providers.

##### Provider investment

Providers expressed their commitment to and enthusiasm for the model in the varied ways that they advocated for the programme, often exceeding expectations to make GANC succeed.

They [clinicians] facilitated groups, solved logistical problems, did ‘everything’ that needed to be done, aggressively recruited women, advocated and ‘tapped into every resource.’—Unidentified Clinician Facilitators in the U.S. [50, p 470]

Providers differed in their opinions of whether GANC reduced workload or increased it. While, as identified above, they found that the repetition was decreased and they had more time to dedicate to support, relationship building and in-depth education, learning a new model of care increased the work needed in preparation, particularly at the start of programme implementation [54, 57, 67].

In the beginning, it [GANC] created more work and the atmosphere was chaotic and stressful.—Midwife in the U.S. [52, p 214]

The work described fell into two categories, one involving the mental challenge of facilitation and the other being the logistical effort put into the structural functioning of GANC within a health care organization.

##### Organisational impact

The workload was perceived as more onerous in the presence of organizational barriers (raised in 11 included studies), such as in cases where staffing shortages didn’t allow for a co-facilitator or a provider had to cover intrapartum and antepartum services simultaneously, or there was inadequate administrative buy-in.

Sometimes I felt, like, helter-skelter trying to do everything by doing this by myself, it’s more work than one-on-one care.—Midwife in the U.S. [49, p 695]

In spite of their flexibility, enthusiasm and commitment, some providers experienced real challenges in this model of care. Most of the barriers were organizational: issues around scheduling, staffing, charting and following up labs, lack of support or recognition from colleagues or management, or generalized system dysfunction [54, 61, 63, 67].

So we need one person who coordinates it from [hospital] side. Because there’s so many things to follow-up, to prepare, we need a permanent staff member to continue to organise all of the groups, all of the charts to be prepared, all of the follow up bloods, ultrasound . . . —Midwife in Australia [68, p 419]

These barriers led some providers to make untenable compromises or to abandon the model altogether [67]. One clinician stated, “…the joy of doing groups is gone.” [49, p 695].

With proper institutional support, most providers found the benefits outweighed the challenges, and several providers felt that GANC reduced their workload or made it easier by increasing confidence in women and reducing unnecessary pages or clinic visits [55, 56, 61, 67]. Findings from Rwanda and the reflection of an Australian midwife indicate that the workload is more manageable when providers have more autonomy over their scheduling in GANC, as with case-loading models [60, 62].

[Group care] adds to our workload as others have said, but I am lucky because it is me who plans the work to be done. Therefore I allot enough time to it;—Nurse/Midwife in Rwanda [60, p 11]

Adequate training in the model and facilitation skills was routinely appreciated by providers facilitating GANC [52, 53, 57, 61].

Providers noted an improvement in participation and acceptance of group ANC over time. They expressed that conducting group ANC was easy (n = 4) and stressed the importance of using guides and having ongoing training.—Midwives and CHW in Nepal [61, p 10].

##### Value return for providers

The overall experience of providers with GANC as reported in the literature was a positive one across a wide variety of contexts and countries, from busy urban clinics to rural low-risk practices. A majority of included providers stated the benefits associated with the programme generally outweighed any additional associated workload. Midwives, physicians, nurses and educators all reported enjoying this type of care delivery model. Speaking specifically about the experience of facilitating GANC, the words ‘joy”, “fun”, “meaningful” were used repeatedly [50, 52, 67].

Group care was for me, a rewarding, enjoyable and far more effective way in engaging with women and families and to meet their educational support needs. I miss ‘my’ women and students greatly.—Midwife in Australia [62, p 89]

This Ibaruke Neza [group ANC/PNC] program which is carried out in the groups made me like my job. Why is that? Clients have lovely and friendly interactions with nurses, they feel at ease when talking with them.—Nurse/Midwife in Rwanda [60, p 10]

## Discussion

The aim of this review was to examine the experiences of health care providers facilitating GANC. The review resulted in three major themes: (1) Giving women the care they want and need; (2) Building skills and relationships; (3) Value proposition of GANC. While the included studies reflected heterogeneity of origin and methodology, there was notable concordance of experience across country and health care organizations. In all three thematic areas, data from high-income and LMICs were represented. The experience of giving women the care that providers feel they want and need was valued by GANC facilitators in every country context. Providers reported building skills and relationships in Ghana and the U.K. [55, 67]. The thematic question of the value proposition of GANC was addressed by participants in rural Nepal and in the urban United States [50, 61]. The parallels reflected in these studies pertained to negative as well as positive experiences. Providers from numerous countries experienced anxiety around the facilitative component of group care and the organizational challenges around implementation of a new model of care [50, 52, 60, 64, 67]. A key finding of this review was that, by and large, GANC offered providers a satisfying option for maternity care providers to provide the kind of quality antenatal care they feel is best for women while simultaneously allowing them to develop their professional role.

Under the theme of providing care that women want, the subthemes the richer use of time, more depth in the time allotted, more personalized care, more supportive care and continuity of care are supported in the Cochrane review on uptake and provision of antenatal care [69]. The experiences of time and continuity in GANC models likely engender the ability to offer more personalized, supportive care, as this has also been reflected in research around case-loading midwifery models which also increase continuity of care and thus time with the same provider [70]. Case-loading time is described as ‘purposeful, flexible, uncertain and personalized’ [71]. These same words could easily be used to describe the facilitating providers’ plan for each GANC session. While case-loading research demonstrates why close relationships between women and providers are important, the finding from this review that providers experienced group care as ‘individualised’ [67, p 61] is surprising and somewhat counterintuitive and warrants further study.

While the studies included in this review do not specifically address the question of whether or not providers experienced the groups as personally supportive, the findings of provider comfort and ease, in tandem with increased autonomy for the women and the providers, suggest ways in which GANC models could be protective of provider wellbeing. The facilitative nature of GANC may allow midwives to develop more of the relational reciprocity with women that many midwives are seeking [72]. Burnout among health care professionals has been linked to lower quality care, lower patient satisfaction and high staff turnover, which is of particular concern amidst global maternity care provider shortages [40, 73]. Although there is a burgeoning body of literature around maternity care professionals’ experiences of burnout and birth trauma, there is little evidence around the impact of antenatal care delivery on overall professional wellbeing [74].

Similarly, lack of opportunities around skill building and professional development have been highlighted as contributing to dissatisfaction among maternity care providers globally [75]. The findings around the theme of building skills and relationships in this review support the concept of role development as a contributor to professional satisfaction. The subthemes of independence/autonomy, provider role development and hierarchy shifts suggest GANC offers new avenues for meeting WHO recommendations on task shifting in maternity care while also fulfilling expressed provider desires around greater professional self-determination [1, 75]. It has been suggested that one contributor to disrespectful care of women in sub-Saharan Africa may be a desire by disempowered midwives to maintain status through enforcing hierarchies [76]. In contrast, the findings in this review from LMICs suggest that providers facilitating GANC found the dissolution of hierarchies a positive experience for providers, raising research questions on the possible impacts of GANC on disrespect and abuse in maternity care.

The findings under the third theme, Value Proposition of GANC, raise important questions about the agency of individual providers (even very committed ones) to affect health care delivery systems.. It supports recent findings from research that suggest that whilst successful implementation of group care models certainly need providers to be enthusiastic and satisfied, without systemic organizational-level planning and support, sustainability is threatened [29, 77]. Although this review has found a surprising number of similarities across country contexts, there is little doubt that, just as the providers in this review benefit from understanding and responding to the needs of the individual women in their groups, organizations implementing GANC would benefit from understanding and responding to the individual needs of their facilitating providers. This review did not have enough data to conduct a sub-analysis of provider experiences by provider type, so it is uncertain whether midwives’ experiences were notably similar to, or different from, those of physicians, nurses or community health workers.

The strengths of this review lie in the robust nature of the systematic search and the quality, quantity, and diversity of the nature of the papers that met the inclusion criteria. Limitations include methodologic considerations of the included studies, such as a lack of clarity around defining the roles of study participants, a lack of researcher reflexivity in some included studies, and the possible impact of social desirability bias on the findings from interviews and focus group discussions evaluating GANC interventions. This is of particular concern in low-income country contexts where programme implementation may be dependent on external non-governmental organizational funding and participants may be wary that negative feedback could result in economic or political repercussions. The first author has experience as a midwife in GANC; in order to minimize associated biases, the researcher used reflexivity, disconfirming analysis and a diverse research team in analysis and synthesis.

### Areas for further research

The review demonstrated that whilst there is now a significant body of research that includes experiences of providers facilitating GANC, most of the findings are drawn from research in the context of pilot feasibility trials. The experiences of the providers obtained in these pilots reflect the particular needs of new programme implementation and evaluation research and may differ significantly from the views of providers who have been delivering GANC in systems where it has become a more routine health care option. The effort and change involved in undertaking a completely new way of working in antenatal care will almost certainly yield different perspectives than those to be found among professionals who have adapted and integrated this complex intervention into their daily working lives. Further research in this area is therefore warranted to get a more complete picture of the provider experience of integration of GANC into a health care system.

## Conclusion

This review examined health care providers’ experiences of facilitating GANC. The review demonstrates benefits for providers of working within GANC models, specifically experiences of delivering responsive high-quality care that they feel is valued by women and is satisfying professionally. Skill building and interprofessional collaboration offer additional areas for provider growth. Fulfilling the global recommendations for the implementation of GANC as a viable alternative to standard antenatal care will continue to require the input and voice of experienced providers to successfully reap the benefits for women, families and the providers and systems themselves.

## Supplementary Information

**Additional file 1:** Search terms for healthcare providers’ experiences of GANC.

## Data Availability

Any supporting data not included in this article and supporting documents can be requested by contacting the corresponding author.

## Abbreviations

GANC: Group antenatal care
LMICs: Low- and middle-income countries
WHO: World Health Organization
