# Supplementary material for: A systematic review of providers’ experiences of facilitating group antenatal care
Source: Reprod Health. 2021 Sep 7;18:180. doi: 10.1186/s12978-021-01200-0 (PMC8425020; doi:10.1186/s12978-021-01200-0)
Supplement: Supplementary file 1 — Additional file 1: Search terms for healthcare providers’ experiences of GANC. [file 12978_2021_1200_MOESM1_ESM.docx]

**Additional File 1: Search Terms for Healthcare Providers’ Experiences of GANC**

|  |  | MeSH headings | Tiab keyword |
| --- | --- | --- | --- |
| **Group Antenatal Care Terms** | Antenatal Care | (MH "Prenatal Care") OR (MH "Prenatal Diagnosis") OR (MH "Perinatal Care") OR (MH "Maternal Health Services") OR (MH "Obstetric Nursing") OR (MH "Parenting Education") | (prenatal OR "pre natal" OR antenatal OR "ante natal" OR perinatal OR "peri natal")  W1 (care OR control OR education OR intervention) |
|  | Group Care | (MH "Group Processes")  OR (MH "Peer Group") | Group education OR group class* OR group screening* OR group assessment* OR group checkup* OR group check-up* OR group check up* OR Group Family Nurse Partnership* OR gFNP |
|  | Group Antenatal Care |  | AB "CenteringPregnancy" or "Centering Pregnancy" OR (group antenatal OR group prenatal OR group ante-natal OR group prenatal)  W1 (care OR education OR class* OR assessment* OR checkup* OR check-up* or check up*) |
|  | 1 AND 2 OR 3 |  |  |
| **Healthcare Providers’ Experiences** | Health Professionals | MH "Health Personnel") OR (MH "Allied Health Personnel") OR  (MH "Community Health Workers") OR (MH "Medical Staff") OR (MH "Midwives") OR (MH "Nurses") OR  (MH "Physicians") OR  (MH "Attitude of Health Personnel") OR (MH "Midwife Attitudes") OR (MH "Nurse Attitudes") OR (MH "Physician Assistant Attitudes") OR  (MH "Work Experiences") | AB physician* OR  midwi* OR nurse* OR "healthcare provider" OR "healthcare providers |
|  | Experiences |  | AB (physician* OR  midwi* OR nurse* OR "healthcare provider" OR "healthcare providers") W3 (view* OR perspective* OR experience*) |
|  | 5 OR 6 |  |  |
| **Full Search** | 4 AND 7 |  |  |
